# A multi-environment framework to evaluate the adaptation of wheat (*Triticum aestivum*) to heat stress

**DOI:** 10.1007/s00122-021-04024-5

**Published:** 2022-01-20

**Authors:** Paul Telfer, James Edwards, Julian Taylor, Jason A. Able, Haydn Kuchel

**Affiliations:** 1 Australian Grain Technologies, 20 Leitch Road, Roseworthy, SA 5371 Australia; 2 School of Agriculture, Food and Wine, The University of Adelaide, Waite Campus, PMB 1 Glen Osmond, Adelaide, SA 5064 Australia

## Abstract

**Key message:**

Assessing adaptation to abiotic stresses such as high temperature conditions across multiple environments presents opportunities for breeders to target selection for broad adaptation and specific adaptation.

**Abstract:**

Adaptation of wheat to heat stress is an important component of adaptation in variable climates such as the cereal producing areas of Australia. However, in variable climates stress conditions may not be present in every season or are present to varying degrees, at different times during the season. Such conditions complicate plant breeders’ ability to select for adaptation to abiotic stress. This study presents a framework for the assessment of the genetic basis of adaptation to heat stress conditions with improved relevance to breeders’ selection objectives. The framework was applied here with the evaluation of 1225 doubled haploid lines from five populations across six environments (three environments selected for contrasting temperature stress conditions during anthesis and grain fill periods, over two consecutive seasons), using regionally best practice planting times to evaluate the role of heat stress conditions in genotype adaptation. Temperature co-variates were determined for each genotype, in each environment, for the anthesis and grain fill periods. Genome-wide QTL analysis identified performance QTL for stable effects across all environments, and QTL that illustrated responsiveness to heat stress conditions across the sampled environments. A total of 199 QTL were identified, including 60 performance QTL, and 139 responsiveness QTL. Of the identified QTL, 99 occurred independent of the 21 anthesis date QTL identified. Assessing adaptation to heat stress conditions as the combination of performance and responsiveness offers breeders opportunities to select for grain yield stability across a range of environments, as well as genotypes with higher relative yield in stress conditions.

**Supplementary Information:**

The online version contains supplementary material available at 10.1007/s00122-021-04024-5.

## Introduction

Many regions throughout the world experience heat, drought, and frost stress that limit crop production. In the Mediterranean-type climates of southern Australia, heat stress conditions during the sensitive crop development stages of anthesis and early grain filling are common (Zheng et al. 2012), often co-occurring with other abiotic stresses such as drought and high wind (Machado and Paulsen 2001; Shah and Paulsen 2003). In southern Australia, heat stress conditions during anthesis and early grain fill are typically short periods, of up to a few days in length, with daily maximum temperatures in excess of 35 °C accompanied with winds in excess of 40 km h^−1^ (Alexander et al. 2010; Talukder et al. 2013). The impacts of such stress events can be significant, with Kuchel et al. (2007a) and Bennett et al. (2012b) reporting yield loss of up to 187 kg ha^−1^ for every one-degree increase in average temperature during anthesis and grain fill in field experiments conducted across southern Australia. This was confirmed by Telfer et al. (2018), who identified a reduction in grain yield of 161 kg ha^−1^ for each day with a maximum temperature in excess of 30 °C during grain fill, and a reduction of 302 kg ha^−1^ for each day with maximum temperature in excess of 30 °C during anthesis.

Stress conditions, like those described, impact negatively on a range of developmental stages and physiological processes (Wahid et al. 2007). Plant mechanisms that manage antioxidants, heat shock proteins, maintenance of cell membrane stability, and maintenance of protein stability and function often underpin a plant's ability to cope with stress (Dolferus et al. 2011; Wahid et al. 2007). In the field, heat stress results in reduced pollen viability and reduced seed set when it occurs during anthesis (Dolferus et al. 2011; Saini et al. 1999). Stress during grain filling leads to reduced starch and protein accumulation (Bhullar and Jenner 1985; Zahedi et al. 2004), accelerated plant development, premature leaf senescence, and reduced photosynthetic rate and capacity (Stone and Nicolas 1995; Tewolde et al. 2006), which ultimately reduces grain size (Sharma et al. 2008; Stone and Nicolas 1995; Talukder et al. 2014a; Wardlaw 1994) and grain yield (Talukder et al. 2014a; Tewolde et al. 2006).

Tolerance to heat stress in wheat has been previously reported. This includes the Australian wheat varieties Halberd (Hays et al. 2007) and Gladius (Fleury et al. 2010; Talukder et al. 2014a), as well as breeding and research line, RAC875 (Bennett et al. 2012a; Izanloo et al. 2008). This was also confirmed by Telfer et al. (2018) who showed that Halberd expressed tolerance to heat stress during flowering, and Gladius and RAC875 expressed tolerance to heat stress during grain filling. Additionally, Gladius and RAC875 have been reported as drought-tolerant (Bennett et al. 2012a; Fleury et al. 2010; Izanloo et al. 2008; Shirdelmoghanloo et al. 2016a).

Various methods have been used to evaluate performance under heat stress conditions, using controlled environments or in-field conditions, including Telfer et al. (2018) who compared a controlled environment assay and a field assay for the evaluation of adaption to heat stress conditions. Controlled environment conditions offer many advantages, including ensuring a consistent and repeatable stress environment and being able to manage a range of confounding factors that may be present in field conditions. The oftentimes confounding effects of maturity can be managed using controlled environments, in contrast to field experiments where material that develops at a different rate may be exposed to different temperature stress conditions making comparisons challenging. Despite the advantages of controlled environments, validation is required under representative field conditions. To ensure a high incidence of heat stress conditions, delayed sowing has been a commonly used methodology to ensure that sensitive developmental stages coincide with heat stress conditions typically experienced later in the season (Bennett et al. 2012b; Esten Mason et al. 2013; Pinto et al. 2010; Reynolds et al. 2007; Sadras et al. 2015). Unfortunately, in this system plants are exposed to growing conditions that are not representative of agronomic practices employed by grain producers, such as longer photoperiod and altered plant available water (Sadras et al. 2015). With phenological development, a primary driver of adaptation, and development rate interacting strongly with sowing date, assessments of heat stress adaptation using sowing date variation are consequently confounded, precluding clear conclusions being drawn from such studies.

Field evaluation of heat stress adaptation has also been carried out using portable heat chambers in the field to induce heat stress treatments (Alexander et al. 2010; Talukder et al. 2013; Thistlethwaite et al. 2020). Such methodology provides the relevance of a field trial, being managed to agronomic best practice, with the added benefit of controlled environment evaluation through consistent heat stress treatments and fewer confounding factors arising from variation in phenology. The physical encumbrance of handling heat chambers in the field, however, largely limits such systems to small exploratory experiments with few genotypes.

Telfer et al. (2018) previously discussed an alternative approach whereby the heat stress tolerance of wheat germplasm was evaluated across a multi-environment (contrasting for heat stress) study. Such a system enables the material to be grown in representative growing environments using regional best practice agronomy and using the natural variation in temperature across the environments to evaluate genotype response to heat stress. This provides the opportunity to evaluate adaptation to heat stress conditions in large-scale breeding trials across a range of environments, subsequently enabling the identification and validation of genetic loci using genetic mapping populations. Kuchel et al. (2007a) also reported similar results, having evaluated doubled haploid bi-parental material across multiple representative environments. In that study, variable expression of QTL for grain yield, and associated traits, were attributable to environmental factors such as temperature and rainfall conditions varying across the environments sampled. Similarly, Tura et al. (2020) was able to identify QTL-by-environment interactions for grain yield and related traits.

A number of studies have identified QTL for adaptation to heat stress conditions that potentially confer improved grain yield (Bennett et al. 2012b; Bhusal et al. 2017; El Hassouni et al. 2019; Esten Mason et al. 2013; Hassan et al. 2018; Liu et al. 2019; Paliwal et al. 2012; Pinto et al. 2010, 2016; Tadesse et al. 2019; Tahmasebi et al. 2017; Vijayalakshmi et al. 2010), grain size (Ali et al. 2013; Bennett et al. 2012b; Bhusal et al. 2017; Esten Mason et al. 2010; Guan et al. 2018; Liu et al. 2019; Mason et al. 2011; Mohammadi et al. 2008; Paliwal et al. 2012; Pinto et al. 2010, 2016; Shirdelmoghanloo 2014; Shirdelmoghanloo et al. 2016b; Tadesse et al. 2019; Tahmasebi et al. 2017), grain number (Bhusal et al. 2017; El Hassouni et al. 2019; Esten Mason et al. 2010; Guan et al. 2018; Liu et al. 2019; Mason et al. 2011; Pinto et al. 2010, 2016; Sharma et al. 2016; Tahmasebi et al. 2017; Telfer et al. 2021), grain fill rate (Paliwal et al. 2012; Pinto et al. 2016; Sharma et al. 2016; Shirdelmoghanloo 2014), harvest index (El Hassouni et al. 2019; Shirdelmoghanloo 2014), senescence rate (Pinto et al. 2016; Shirdelmoghanloo 2014; Vijayalakshmi et al. 2010) and maturation rate (Bhusal et al. 2017; Paliwal et al. 2012; Shirdelmoghanloo 2014). QTL for physiological traits potentially related to heat stress tolerance have also been identified, including leaf chlorophyll content (Ali et al. 2013; Liu et al. 2019; Maulana et al. 2018; Pinto et al. 2010, 2016; Shirdelmoghanloo 2014; Tahmasebi et al. 2017; Talukder et al. 2014b; Vijayalakshmi et al. 2010), canopy temperature (Ali et al. 2013; Bennett et al. 2012b; Liu et al. 2019; Paliwal et al. 2012; Pinto et al. 2010, 2016), photosystem II efficiency (Fv/Mv) (Hassan et al. 2018; Sharma et al. 2017; Vijayalakshmi et al. 2010), NDVI (Liu et al. 2019; Pinto et al. 2010, 2016) and membrane damage (Hassan et al. 2018; Talukder et al. 2014b). These studies have primarily been undertaken using delayed sowing systems in the field (Bennett et al. 2012b; Bhusal et al. 2017; Esten Mason et al. 2013; Hassan et al. 2018; Liu et al. 2019; Paliwal et al. 2012; Pinto et al. 2010, 2016; Sharma et al. 2016; Tahmasebi et al. 2017) or in controlled environment assays (Mason et al. 2011; Maulana et al. 2018; Mohammadi et al. 2008; Shirdelmoghanloo 2014; Talukder et al. 2014b; Telfer et al. 2021; Vijayalakshmi et al. 2010).

As discussed by Lemerle et al. (2006), tolerance has previously been defined as the difference in trait expression between an unstressed control and a stressed treatment. Usually, there is a strong correlation between trait expression in both stressed and unstressed conditions, making this a potentially misleading definition. Lemerle et al. (2006) and Dolferus et al. (2019) further defined tolerance as a positive deviation from the expected response between the stressed and unstressed treatments. As discussed by Telfer et al. (2021), this deviation from the expected response under stress conditions is more accurately interpreted as responsiveness to stress conditions and should be considered as an independent factor in adaptation in combination with performance value across all conditions.

This study builds on the research carried out by Telfer et al. (2021), by applying a framework for the evaluation of the genetic basis of adaptation to heat stress conditions in representative field conditions, and considers adaptation as the combination of performance and responsiveness. Telfer et al. (2021) identified QTL for performance and heat stress responsiveness in controlled environment conditions. This study uses the same populations to explore adaptation to heat stress conditions in representative field conditions. Here, QTL are discussed in relation to their role in adaptation to heat stress conditions as experienced in Mediterranean-type environments of southern Australia, and their relevance to breeding objectives.

## Materials and methods

### Germplasm and genotyping

Eight doubled haploid populations were used to evaluate adaptation to heat stress conditions in Mediterranean-type environments of southern Australia, and to identify QTL for grain yield and related traits across multiple representative environments. The populations used are described by Telfer et al. (2021) and are summarised in Table 1. In brief, these were developed to encompass historical germplasm pools representative of the Australian breeding germplasm pool, as well as to evaluate potential novel sources of heat stress tolerance identified in a heat stress focused identification of germplasm strategy (FIGS) set as mentioned by Telfer et al. (2021). All lines evaluated in this study from each population were genotyped using a custom Axiom™ Affymetrix array containing 18,101 SNP markers as described by Norman et al. (2017). Further, the linkage maps used for QTL analysis (Supplementary Table 1) were created through the R statistical environment (R Core Team 2018) using a combination of the R/qtl (Broman and Sen 2009; Broman and Wu 2015) and R/ASMap (Taylor and Butler 2017) package (Norman et al. 2017), resulting in excess of 5000 polymorphic markers for each population (exact numbers shown in Table 1). To prepare the linkage maps for analysis, the functionality of the WGAIM R package (Taylor and Verbyla 2011) was used to numerically encode the alleles (AA = 1, BB = − 1), impute missing values using the rules of Martínez and Curnow (1994) and generate unique interval markers using Verbyla et al. (2007).

**Table 1:** The DH populations evaluated in this study, summarising population parents and genetic map details

Population name	Pedigree	No. lines in Map	No. polymorphic SNP markers	No. Unique positions	Genetic length (cM)	Mean interval*
MG	Mace/Gladius	176	5047	1429	3009	2.1
SM	Scout/Mace	226	4950	1360	3030	2.2
SG	Scout/Gladius	369	5143	1761	2998	1.7
RG	RAC1548/Gladius	132	5133	1183	3055	2.6
L2G	AUS17840/Gladius	124	5514	1132	3144	2.8

^*^Mean interval (cM) between unique map positions

### Experimental design and plot management

The methodology described by Telfer et al. (2018) to evaluate adaptation to heat stress conditions under representative field conditions across multiple environments was used as the basis for this study. Genetic material was evaluated at three cereal producing locations in South Australia; Angas Valley, Roseworthy, and Winulta, across two seasons in 2015 and 2016, totalling six environments (the details of each experiment are shown in Table 2). These environments were targeted to achieve a range in heat stress conditions, with Winulta having a maritime climate with relatively mild conditions during anthesis and grain filling compared to the inland site of Angas Valley which typically has warmer conditions. Roseworthy historically is intermediate in anthesis and grain fill conditions as is demonstrated by the temperature conditions during anthesis and grain filling in Table 2.

**Table 2:** The field experiments conducted as a part of the study. Summarised by location, the populations and number of lines included in each experiment, experiment dimensions, sowing date, mean anthesis date for each experiment, and mean maximum daily temperature, number of days > 30 °C, number of days > 35 °C during anthesis and grain fill

Experiment	Year	Population	Location	GPS position	Plots	Columns	Rows	Reps	DH Genotypes	Check Genotypes	Sowing Date	Mean Anthesis Date (Degree days sowing to anthesis)	Mean Grain Yield kgha^−1^	Mean Growing Season (May—Oct) Rainfall (mm)	Mean Anthesis Average Maximum Temperature (°C)	Mean Anthesis Number of Days > 30 °C	Mean Grain Fill Average Maximum Temperature (°C)	Mean Grain Fill Number of Days > 30 °C	Mean Grain Fill Number of Days > 35 °C
ANHGSM151	2015	MG, SM, SG	Angas Valley	− 34.75, 139.27	1296	12	108	1.4	922	7	15 May 2015	1451	2277	102	24.3	5.0	31.9	16.7	7.9
ANHGSM161	2016	MG, SM, SG	Angas Valley	− 34.70, 139.25	336	24	14	1.37	245	7	1 June 2016	1511	3379	221	20.8	2.0	26.0	8.8	0.9
RSHGSM152	2015	MG, SM, SG	Roseworthy	− 34.51, 138.68	1296	12	108	1.4	922	7	21 May 2015	1453	2843	190	24.2	4.2	30.6	10.8	6.0
RSHGSM162	2016	MG, SM, SG	Roseworthy	− 34.50, 138.68	336	24	14	1.37	245	7	15 May 2016	1511	6203	480	20.9	0.0	23.1	1.8	0.0
WTHGSM153	2015	MG, SM, SG	Winulta	− 34.30, 137.90	1296	12	108	1.4	922	7	12 May 2015	1458	2591	208	22.8	3.0	28.1	11.1	4.5
WTHGSM163	2016	MG, SM, SG	Winulta	− 34.26, 137.90	336	24	14	1.37	245	7	18 May 2016	1510	6972	381	19.5	0.0	23.2	2.9	0.0
ANHX32151	2015	L2G	Angas Valley	− 34.75, 139.27	216	12	18	1.52	142	8	15 May 2015	1379	1644	102	22.5	3.0	31.9	16.8	7.6
ANHX32161	2016	L2G	Angas Valley	− 34.70, 139.25	144	24	6	1.67	86	9	1 June 2016	1555	3298	221	21.5	2.6	26.5	9.3	1.4
RSHX32152	2015	L2G	Roseworthy	− 34.51, 138.68	216	12	18	1.52	142	8	22 May 2015	1387	2489	190	22.8	3.0	30.8	12.2	7.2
RSHX32162	2016	L2G	Roseworthy	− 34.50, 138.68	144	24	6	1.67	86	9	15 May 2016	1556	6226	480	20.8	0.0	23.6	2.5	0.0
WTHX32153	2015	L2G	Winulta	− 34.30, 137.90	216	12	18	1.52	142	8	12 May 2015	1382	2447	208	21.5	1.3	28.0	11.5	5.1
WTHX32163	2016	L2G	Winulta	− 34.26, 137. 90	144	24	6	1.67	86	9	18 May 2016	1552	6734	381	19.6	0.0	23.5	3.3	0.3
ANHX4151	2015	RG	Angas Valley	− 34.75, 139.27	240	12	20	1.49	161	8	15 May 2015	1380	2181	102	22.4	2.8	32.2	17.1	7.8
ANHX4161	2016	RG	Angas Valley	− 34.70, 139.25	192	24	8	1.62	117	8	1 June 2016	1565	3540	221	21.6	2.7	26.6	9.3	1.5
RSHX4152	2015	RG	Roseworthy	− 34.51, 138.68	240	12	20	1.49	161	8	22 May 2015	1379	2738	190	22.5	2.5	31.0	12.4	7.5
RSHX4162	2016	RG	Roseworthy	− 34.50, 138.68	192	24	8	1.62	117	8	15 May 2016	1566	7202	480	20.7	0.0	23.8	2.6	0.0
WTHX4153	2015	RG	Winulta	− 34.30, 137.90	240	12	20	1.49	161	8	12 May 2015	1381	3043	208	21.4	1.1	27.9	11.7	5.1
WTHX4163	2016	RG	Winulta	− 34.26, 137.90	192	24	8	1.62	117	8	18 May 2016	1567	7903	381	19.6	0.0	23.7	3.4	0.4

The 2015 GSM experiments consisted of 154 MG, 203 SM and 360 SG lines. In 2016, there were 42 MG, 44 SM and 92 SG lines. In each year, the remaining plots in each experiment were made up of material not used in the analysis for this study

Populations were assigned to separate experiments except for the Mace/Gladius (MG), Scout/Mace (SM), and Scout/Gladius (SG) populations, which due to common parentage across the populations, were grouped and referred to as the GSM populations. Fewer lines were evaluated in 2016 compared to 2015. For 2016, entries were a subset of the 2015 entries selected to remove extreme maturity types as identified in the 2015 season. Lines with other extreme adverse phenotypes such as those with very tall plant heights were also removed. No direct selection occurred for grain yield and physical grain quality attributes which were the focus of this study. Field plots within experiments were sown in a grid format, and the experiment details are shown in Table 2. The experimental design used the principles of partially replicated design as discussed by Cullis et al. (2006), with each doubled haploid line in each population present in each environment and each doubled haploid line replicated in one of the three environments of that year. Consequently, doubled haploid lines were replicated on average 1.33 times at each environment, with check varieties (doubled haploid parents and locally adapted varieties) fully replicated in each environment (Table 2).

Within each experiment, plots were sown 1.32 m wide by 5 m long, with plots reduced to a length of 3.2 m before anthesis using herbicide. Each experiment was sown to achieve 200 seeds m^−2^. Field experiments were managed using regional best practice agronomy, encompassing sowing and harvest times, crop nutrition, and pest (weed, insect, and fungal) management.

### Phenotyping

At Roseworthy, in each year of the study, each plot was assessed to determine spike emergence date (50% of spikes fully emerged from the flag leaf sheath) of each plot. Along with temperature data collected at each environment (explained in further detail later), a degree-day model (Sadras and Monzon 2006) was used to estimate the anthesis date of each line in each environment, using daily mean temperature > 0 °C as the base temperature for plant development. Relative maturity observations were also made at each location in each year of the study to confirm the accuracy of the modelled anthesis date as described by Telfer et al. (2018).

All experiments were harvested and weighed after reaching physiological maturity, to determine grain yield (kg ha^−1^). The grain was further evaluated for screenings percentage assessed by determining the weight of matter that passes through a 2 mm slotted sieve (Grain Trade Australia 2020), and test weight (TWT, kg hl^−1^), assessed by weighing a 500 mL sample measured using a chrondrometer (Grain Trade Australia 2020).

### Climatic co-variates to describe crop stress conditions

Temperature data were collected in each environment for the duration of the growing season using a single factory-calibrated temperature logger (TinyTag™ Talk2), logging on half-hourly intervals. The temperature logger was situated immediately adjacent to the block of experiments all situated adjacent to each other in each location, at 1 m in height to replicate approximate crop height at anthesis and grain fill.

Temperature observations were used to calculate climatic co-variates to describe the temperature conditions experienced by each line in each environment during the key developmental periods, anthesis (300°Cd before anthesis to 100°Cd post-anthesis) and grain filling (100°Cd to 600°Cd post anthesis). Climatic co-variates calculated for each line in each environment included average maximum temperature (°C), number of days > 30 °C and number of days > 35 °C, during both anthesis and grain filling (Dreccer et al. 2008; Telfer et al. 2018). Growing season rainfall (total cumulative rainfall (mm) from the start of May to the end of October) was also recorded in each environment. In the 2015 season, this was taken from the nearest Australian Bureau of Meteorology (BOM) weather station; Angas Valley sourced from Cambrai BOM station (No. 024513), Roseworthy sourced from Roseworthy BOM station (No. 023021) and Winulta sourced from Ardrossan BOM station (No. 022021)). In the 2016 season, this was measured on-site using a factory-calibrated Davis^R^ Vantage Vue Weather Station situated adjacent to the experiments logging at 15-min intervals for the duration of the experiment in each environment. The mean climatic co-variate observed across all lines in each experiment is shown in Table 2. The length of the anthesis period ranged from 29 to 36 days, and the grain fill period ranged from 24 to 36 days depending on the population and the environment in which they were grown.

### Statistical methods—Baseline model

For each of the populations, assume a set of $r$ progeny varieties were sown in $t$ environments and let ${\varvec{y}} = \left( {{\varvec{y}}_{1}^{T} \ldots {\varvec{y}}_{t}^{T} } \right)^{T}$ be a vector of observed responses such as grain yield, screenings or TWT. The response was then analysed using a baseline multi-environment linear mixed model (ME-LMM) of the form

1 $${\varvec{y}} = {X\tau } + {\varvec{Zu}} + {\varvec{Z}}_{g} {\varvec{u}}_{g} + {\varvec{e}}$$

where ${X\tau }$ was a fixed component containing a vector of fixed effects for estimating experiment means as well as an overall mean for the population, parents, and controls specific to each environment. ${\varvec{Zu}}$ was a random component consisting of effects ${\varvec{u}}$ that were conformably partitioned to account for environment specific variation such as aspects of the experimental designs including blocks or replicates, and also variation due to potential extraneous nonlinear trends potentially existing across the row or column of the experiment. To further improve the flexibility of the ME-LMM to model local spatial variability, the residuals were partitioned to ${\varvec{e}} = \left( {{\varvec{e}}_{1}^{T} \ldots {\varvec{e}}_{t}^{T} } \right)^{T} { }$ where the residuals within the $j$ th environment were assumed to be distributed ${\varvec{e}}_{j} \user2{ }\sim \user2{ }N\left( {0,\user2{ }\sigma_{j}^{2} {\varvec{R}}_{j} } \right)$ with ${\varvec{R}}_{j}$ parameterised as a separable auto-regressive structure of order one in the Row and Column direction. The important focus of this ME-LMM is the random genotype-by-environment interaction term ${\varvec{Z}}_{g} {\varvec{u}}_{g}$, with effects ${\varvec{u}}_{g}$ of length $r \times t$ and indicator matrix ${\varvec{Z}}_{g}$ that maps the genotypes to the plots within each experiment. These effects were assumed to be distributed ${\varvec{u}}_{g} \user2{ }\sim \user2{ }N\left( {0,\user2{ }{{\varvec{\Delta}}} \otimes {\varvec{I}}_{r} } \right)\user2{ }$ where ${{\varvec{\Delta}}}$ is a $t \times t$ covariance matrix consisting of environment-specific variances on its diagonal to capture genetic variation of the progeny lines within each environment and covariances on its off diagonals to model the genetic relatedness of the progeny between each pair of environments. When $t$ was moderately large the estimation of ${{\varvec{\Delta}}}$ became problematic and we sought the use of a parsimonious approximation by defining the genotype by environment effects to have a Factor Analytic (FA_*k*_) model (Smith et al. 2001, 2005) with variance ${\mathbf{\Lambda \Lambda }}^{T} + {{\varvec{\Psi}}} \approx {{\varvec{\Delta}}}$ where ${{\varvec{\Lambda}}}$ is $t \times k$ matrix of environment loadings and ${{\varvec{\Psi}}}$ is a diagonal matrix containing environment specific variances. The number of factors $k$ used in the FA model depended on the number of environments in which the population progeny were sown.

### Statistical methods-QTL analysis

For a given population, let ${\varvec{M}}$ be an $r \times p$ matrix of unique interval markers. For each trait from this population, a QTL analysis was conducted using a whole genome scanning approach with two separate runs. In the first run, the focus was to obtain overall performance QTL across environments. Let ${\varvec{m}}_{ij}$ be the $j$ th interval marker in the $i$ th chromosome, then the performance QTL model was an extension of the ME-LMM defined in (1) where the total genetic effects were partitioned as

2 $${\varvec{u}}_{g} = {\varvec{m}}_{ij} a_{ij} + \user2{ u}_{a} + {\varvec{u}}_{p}$$

In this extended genetic model, $a_{ij}$ represents the main or overall performance effect of the interval marker ${\varvec{m}}_{ij}$ across the $t$ environments. We used the strategy of Rincent et al. (2014) for improving the power of detecting significant marker or marker by climatic interaction effects by including additional genomic and residual genetic terms in (2). Specifically, we include ${\varvec{u}}_{a}$, a vector of random marker based additive genotype by environment effects that are assumed to be distributed ${\varvec{u}}_{a} \user2{ }\sim \user2{ }N\left( {0,\user2{ }{{\varvec{\Delta}}}_{a} \otimes {\varvec{G}}_{ - i} } \right)$ where ${\varvec{G}}_{ - i} = \user2{ M}_{ - i} {\varvec{M}}_{ - i}^{T}$ is an $r \times r$ genomic relationship matrix with the interval markers from the $i$ th chromosome removed and ${{\varvec{\Delta}}}_{a}$ is an $t \times t$ additive genetic covariance matrix. We also include ${\varvec{u}}_{p}$, a vector of random polygenic residual genotype by environment effects that are assumed to be distributed ${\varvec{u}}_{p} \user2{ }\sim \user2{ }N\left( {0,\user2{ }{{\varvec{\Delta}}}_{p} \otimes {\varvec{I}}_{r} } \right)\user2{ }$ where ${{\varvec{\Delta}}}_{p}$ is a $t \times t$ residual genetic covariance matrix. Similar to the previous sections, where necessary, the marker-based additive and residual genotype by environment effects were both parsimoniously approximated by a Factor Analytic model.

In the second run, the focus was on detecting QTL displaying significant responsiveness across the numerical range of the climatic covariate. If ${\varvec{c}}$ is the climatic covariate, then the climate covariate QTL model used an extended ME-LMM with ${\varvec{cm}}_{ij} b_{ij}$ replacing the first term on the right-hand side of (2).

In this new term $b_{ij}$ represents the interval marker by climatic covariate interaction effect or responsiveness effect of the interval marker across environments.

The two QTL models were then used to scan the complete $p$ interval markers across the 21 chromosomes of the wheat genome, and Wald statistics were calculated for each performance and responsiveness effect. Initial effect significance was determined using the thresholding technique of Li and Ji (2005) outlined for QTL ME-LMMs in Bonneau et al. (2013). Interval marker effects above the significance threshold were omitted from further analysis if they were within a window of 30 cM adjacent to an interval marker effect with greater significance. The remaining set of $n_{s}$ significant interval markers were then considered linked to putative QTL and additively included in a final QTL model, using the example of significant performance QTL, was

3 $${\varvec{u}}_{g} = \mathop \sum \limits_{s = 1}^{{n_{s} }} {\varvec{m}}_{s} a_{s} + \user2{ u}_{a}^{*} + {\varvec{u}}_{p}$$

where ${\varvec{u}}_{a}^{*} \user2{ }\sim \user2{ }N\left( {0,\user2{ }{{\varvec{\Delta}}}_{a} \otimes {\varvec{G}}_{ - s} } \right)$ and ${\varvec{G}}_{ - s} = \user2{ M}_{ - s} {\varvec{M}}_{ - s}^{T}$ is the genomic relationship matrix with interval markers excluded if they were within a 30 cM window of the interval markers in the final model. The significant performance and responsiveness QTL were then summarised with their chromosome, genetic distance, LOD score, and additive effect size. To aid in the interpretation of the size of the additive effect for responsiveness, a normalisation was conducted that multiplied each additive effect by the numerical range of the covariate over the environments. The normalised additive effect size was also provided. For each of the QTL, a linked marker was used to identify the RefSeq physical position (Alaux et al. 2018), with the base pair position at the start of the candidate marker sequence. Analysis to identify QTL for anthesis data was only conducted using the described methodology to identify performance QTL, as these data were only collected in the experiments collected at Roseworthy in each year of the study.

### Statistical methods-Computations

Baseline ME-LMMs were fitted using the flexible linear mixed modelling software package ASReml-R (Butler et al. 2018) available in the R statistical computing environment and downloadable through VSNi website from https://www.vsni.co.uk/software/asreml. The ASReml-R package contains a suite of functionality for fitting and diagnosing complex linear mixed models and uses the REML algorithm of Patterson and Thompson (1971) to estimate model parameters. Diagnostic assessment of models was conducted using ASReml-R functions as well as functions from the post-processing package ASExtras available for download from https://mmade.org/. QTL analyses were conducted using the GWASReml R software package available from https://github.com/DrJ001/GWASReml. The package provides functionality for conducting one-stage QTL and GWAS analyses through extensions of the baseline ASReml-R model.

## Results

### QTL identified for performance

**Fig. 1:**
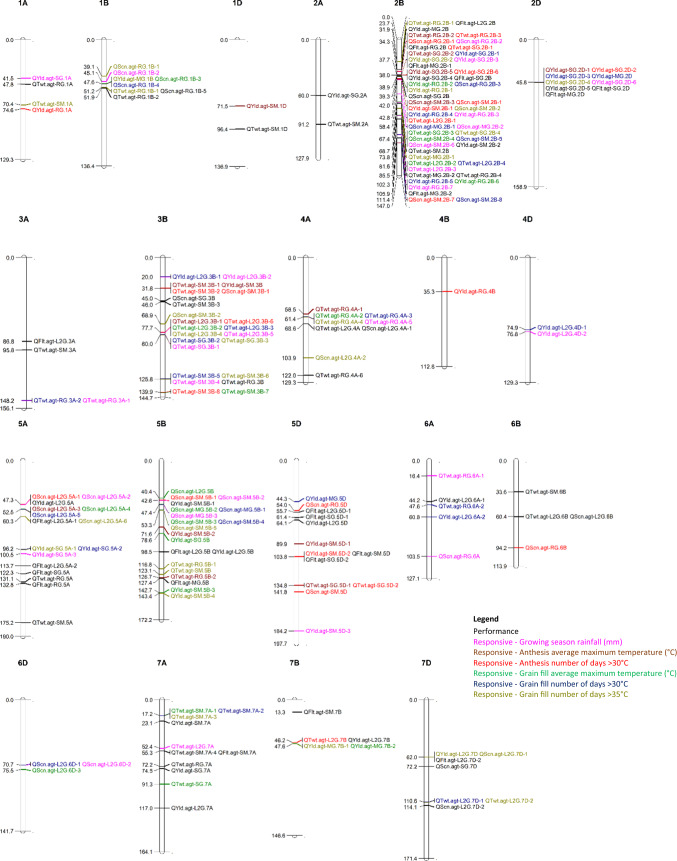
QTL identified for performance and responsiveness to the climatic co-variates measured in each environment mapped against their position (cM) on the consensus map. Colours indicate QTL type and to which climatic co-variate the responsiveness QTL interact

In total, 199 QTL were found in this study, of which 60 were found to be important for performance, with a stable effect across all environments sampled. QTL were spread across all chromosomes except for chromosome 3D, as shown in Fig. 1 (additional details for each QTL identified are shown in Supplementary Table 2). Genetic correlations for each population for each trait across each environment are shown in Supplementary Table 3. Of the 60 QTL for trait performance, 21 were identified for anthesis date, 14 for grain yield, 18 for TWT, and seven for screenings. Performance QTL were identified in all populations, although not for each trait in each population. Grain yield QTL were not identified in the RG population, TWT QTL were not identified in the SG population, while screenings QTL were not identified in the MG and SM populations. Anthesis date QTL were identified in all populations.

### QTL identified for responsiveness to climatic co-variates

A further 139 QTL (Fig. 1 and Supplementary Table 2) were identified that showed a significant interaction with the climatic co-variates measured for each genotype in each environment. These QTL are termed ‘responsive’, indicating that there is variable trait expression in response to the climatic co-variates from each environment. Grain yield accounted for 44 of the QTL for responsiveness identified across all populations and climatic co-variates. For screenings, 43 responsiveness QTL were identified in the MG, SM, RG, and AUS17840/Gladius (L2G) populations, while none were identified in the SG population. A further 52 responsiveness QTL were found for TWT across all populations and all climatic co-variates, except for the MG population where only one responsiveness QTL was found for grain fill days > 35 °C.

### Clusters of QTL

Of the 199 QTL identified, 18 occurred independently of any other QTL identified. The remaining 181 QTL occurred in clusters with one or more other QTL (within 10 cM of the interval associated with other QTL), with up to 27 QTL clustering together. QTL clusters included those characterised as performance QTL as well as responsiveness QTL.

There were 99 QTL that occurred in combination with each other, but which were not associated with QTL for anthesis date. These clusters in many cases combined QTL for performance and responsiveness for a range of climatic co-variates, and a range of traits. However, there were other regions identified where performance QTL only clustered with performance QTL and responsiveness QTL only clustered with responsiveness QTL. The QTL found to not be associated with anthesis date will be discussed in more depth as they offer opportunities to understand adaptation to heat stress conditions independently from anthesis date, which although important, are often considered differently by plant breeders as anthesis date is also an important selection target.

Of the 21 QTL identified for anthesis date, all except one (*QFlt.agt-SM.7B* on chromosome 7B) clustered with other QTL. This included a genomic region on chromosome 2B where anthesis date QTL were identified between 34.2 cM and 38.8 cM at the same position in the RG, MG, and SG populations, and these clustered with 24 other QTL between 29.8 cM and 43.5 cM. Although not specifically mapped in this study, this region likely aligns with the *Ppd-B1* photoperiod gene found on chromosome 2B (Scarth and Law 1983). QTL associated with this cluster were encompassed by each of the populations evaluated, each trait assessed, and each climatic co-variate measured. Similar but smaller clusters were found on chromosome 2D (likely associated with *Ppd-D1* (Law et al. 1978)), chromosome 5B (likely associated with *Vrn-B1* (Iwaki et al. 2002)), chromosome 5D (likely associated with *Vrn-D1* (Law et al. 1976)), as well as chromosomes 7A, 7B and 7D likely associated with the *FT* gene (Bonnin et al. 2008). On chromosome 5A, three anthesis date QTL were found to be clustered with one TWT QTL (*QTwt.agt-RG.5A*), which likely aligns to *Vrn-A1* (Law et al. 1976). These regions will not be discussed in depth as there are well understood associations with crop performance and phenology genes, as well as a strong confounding influence on adaptation to heat stress effect because anthesis date will influence the level of stress experienced.

### Interpreting QTL with performance and responsiveness effects

**Fig. 2:**
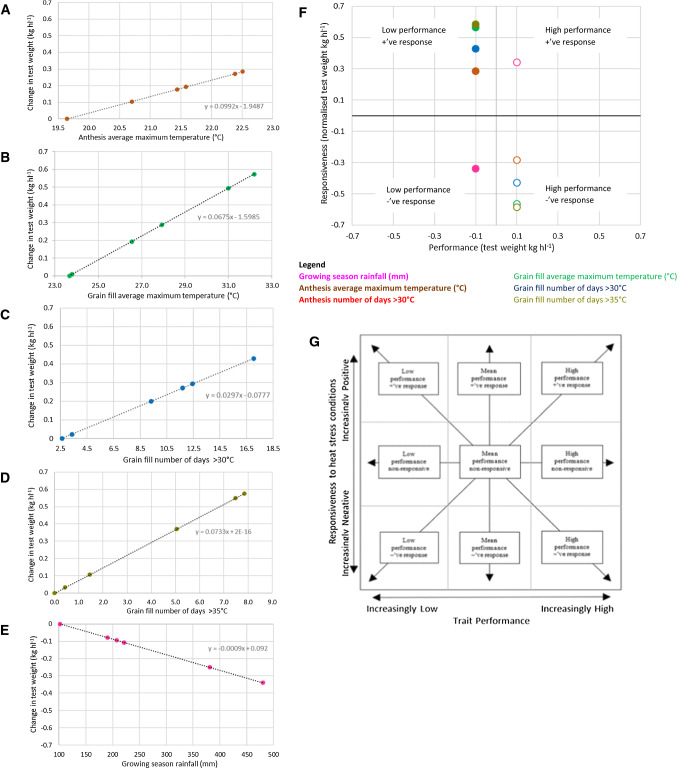
To demonstrate the relationship between performance and responsiveness QTL, an example of TWT responsiveness QTL for various co-variates found to cluster with the TWT performance QTL QTwt.agt-L2G.4A, on chromosome 4A, are shown. For each responsiveness QTL, the additive effect on TWT for the RAC1548 allele is shown for the range observed for each climatic co-variate for; **a** QTwt.agt-RG.4A-1, **b** QTwt.agt-RG.4A-2, **c** QTwt.agt-RG.4A-3, **d **QTwt.agt-RG.4A-4 and **e** QTwt.agt-RG.4A-5. **f** Illustrates the impact on responsiveness by selecting for either the favourable performance allele (Gladius shown by open dots) or the alternative allele (RAC1548 allele represented by closed dots). **f** Can be interpreted within the framework proposed by Telfer et al. (2021) shown in (**g**)

Of the QTL identified, there are examples of performance QTL and responsiveness QTL occurring independently from other QTL, as well as performance QTL and responsiveness QTL occurring at the same position. Adding an additional layer of complexity, these regions also encompassed QTL for various phenotypic traits and response to a range of climatic co-variates. This is demonstrated in Fig. 2a–e, where responsiveness QTL collocating with a performance QTL for TWT on chromosome 4A (*QTwt.agt-L2G.4A*) shows the effect of each responsiveness QTL plotted against the range of each climatic co-variate experienced. Figure 2a–d shows the positive effect of the responsiveness QTL on TWT to increasing anthesis average maximum temperature, grain fill average maximum temperature, number of days > 30 °C, and number of days > 35 °C, as well as TWT decreasing in response to increasing growing season rainfall (Fig. 2e). However, given that these QTL collocated with a performance QTL (*QTwt.agt-L2G.4A*), they need to be considered simultaneously. Figure 2f shows the result of selecting either the high or low-performance allele at this locus and the resulting responsiveness QTL allele that would be selected. In this situation, the increased performance selected by targeting the Gladius allele of the *QTwt.agt-L2G.4A* locus would be associated with a negative response to increasing anthesis average maximum temperature, grain fill average maximum temperature, number of days > 30 °C, and number of days > 35 °C, and a positive response in TWT to increasing growing season rainfall. In other words, selecting for the higher performance allele would also lead to greater sensitivity to hot and dry conditions.

## Discussion

### Assessing the contribution of performance and responsiveness QTL to heat stress adaptation

In the first approach to genetic analysis of heat stress adaptation used in this study, performance QTL were found that had consistent effects across a range of environments in the cereal growing areas of South Australia. In the second approach, regions were identified that interacted with climatic co-variates in their effects on the yield related traits researched herein. This dual approach to genetic analysis allows the framework proposed by Telfer et al. (2021) to be applied to the results of a multi-environment field QTL study. In this framework, the authors propose that attempting to characterise a QTL as tolerant fails to completely describe the nature of the genetic control being exerted by the locus. Telfer et al. (2021) suggested that adaptation to heat stress conditions (and likely other abiotic stresses) is better described as the combination of overall performance and responsiveness to stress conditions. Armed with this understanding, the optimal genetic combination can be selected by breeders for the specific heat stress environment being targeted. Alternatively, selection for QTL with high-performance value and no responsiveness may offer stable adaptation across a broad range of environments.

In this study, several of the performance and responsiveness QTL identified were found to occur collocated or closely located (< 10 cM), to anthesis date QTL identified in this study (Fig. 1 and Supplementary Table 2). Anthesis date is a complex trait determined by several mechanisms: photoperiod, vernalisation, and earliness per se, each controlled by several genes, and is a key component of variety adaptation and crop performance (Eagles et al. 2014; Kuchel et al. 2006). Favourable alleles at these loci are selected by breeders to ensure anthesis occurs at favourable times to minimise exposure to stresses such as frost, drought, and heat while maximising grain yield (Flohr et al. 2017). While this is an important topic, here the discussion is restricted to loci (totalling 116 QTL and described in Supplementary Table 2) that can be selected to improve adaptation to heat stress without affecting time to anthesis.

It was frequently observed that QTL alleles with a negative performance effect responded positively to increasing temperature and negatively to increasing rainfall (the reverse being true, logically, for the alternate allele). This response type is not rare (Telfer et al. 2018) and represents the classic genotype-by-environment response of heterogeneity of variance (Finlay and Wilkinson 1963; Malosetti et al. 2013). This was demonstrated by *QTwt.agt-L2G.4A* a performance QTL for TWT on chromosome 4A (Fig. 2), that collocated with responsiveness QTL for temperature co-variates and growing season rainfall (*QTwt.agt-RG.4A-5*). When considering the parental source of the allele conferring a positive trait effect for a performance QTL effect, it was the opposite allele that conferred positive responsiveness to increasing temperature conditions (and negative response to increasing rainfall). The negative association at the 4A locus between responsiveness to increasing temperature and increasing rainfall is not surprising as high temperature conditions and low rainfall are often associated with stressed environments (Lobell et al. 2015). In this case, breeders can select for either high overall performance, or adaptation to heat and drought stress. When considering the framework proposed in Fig. 2h, this would be classified as a positive response to stressed conditions and low performance value. Additionally, this highlights the correlated nature of climatic co-variates used to describe temperature and water stress as discussed by Telfer et al. (2018). However, this also reinforces the importance of considering adaptation as a whole and considering adaptation to stress conditions during different developmental periods as well as other stress factors such as water stress (considered in this study). The importance of this region for TWT has been highlighted previously by Huang et al. (2006), but also for grain yield (Tura et al. 2020; Yu et al. 2018; Zhang et al. 2018) and grain weight traits (Cui et al. 2014; McCartney et al. 2005; Tahmasebi et al. 2017; Tura et al. 2020; Zhang et al. 2018). Furthermore, studies targeting heat stress adaption have also reported QTL for grain yield in this region (Pinto et al. 2010; Tadesse et al. 2019). The region is likely to be TaCWI (Jiang et al. 2015), located in the centromeric region of chromosome 4A. The commercial varieties and breeding line parents used for the mapping populations have been screened for this gene and Gladius carries the large grain allele, while RAC1548 carries the small grain allele. The results of this study align the large grain allele of Gladius with improved TWT but a negative response to stressed conditions. Interestingly, a screenings performance QTL identified in the L2G population attributed the grain size advantage to AUS17840 rather than Gladius, something that will require further investigation.

An example identified in this study where a performance QTL was positively associated with responsiveness QTL for rainfall and temperature conditions under stressed conditions was found on chromosome 5B in the SM and MG populations. Here a performance QTL for grain yield (*QYld.agt-SM.5B-1*) was identified collocated with a range of responsiveness QTL for temperature co-variates during both anthesis and grain fill, with the Mace parent providing the preferred allele for both performance and responsiveness to increasing temperature. Similarly, Mace also provided the favourable drought (low rainfall) responsive allele at collocated QTL (*QScn.agt-SM.5B-2* and *QScn.agt-MG.5B-3*) associated with screenings. When considering the framework posed in Fig. 2h, this would be classified as positive response with high-performance value. This is a rare combination, but one eagerly sought after by breeders as an opportunity to improve both overall performance and relative performance under heat and drought stress. This region was also described by Bennett et al. (2012b) for grain yield under delayed sowing conditions. Additionally, this region has previously been described for grain yield (Yu et al. 2018) and grain weight traits (Cui et al. 2014; Huang et al. 2003; Ramya et al. 2010; Tsilo et al. 2010; Yu et al. 2018; Zhang et al. 2018).

Alternatively, QTL for responsiveness to increasingly hot conditions not associated with rainfall would allow for the selection of genotypes that can cope with increasing terminal temperature stress conditions and retain performance under a range of rainfall environments. An example of this occurred on chromosome 7A, identified in the SM population, where a performance QTL for grain yield (*QYld.agt-SM.7A*) occurred in a similar location to heat responsive QTL for TWT (*QTwt.agt-SM.7A-1, QTwt.agt-SM.7A-2, and QTwt.agt-SM.7A-3).* Here the Mace parent provided the allele conferring both positive performance and positive responsive to stress. This region appears to align with a grain yield QTL identified in delayed sowing conditions by Bennett et al. (2012b), Pinto et al. (2016) and Hassan et al. (2018), grain number and grain weight per spike by Guan et al. (2018), as well as a leaf chlorophyll content QTL by Talukder et al. (2014b). Additionally, this region has been described in non-heat stress literature for its role in grain yield (Narjesi et al. 2015; Tura et al. 2020; Yu et al. 2018) and thousand grain weight (Cui et al. 2014; Groos et al. 2003; Huang et al. 2004).

### Regions of performance and their role in adaptation

Genetic regions conferring a performance advantage in the absence of responsiveness to climatic conditions may provide breeders opportunities to select for broad adaptation to a range of stressed and unstressed environments. When considered within the framework described in Fig. 2h, this would be classified as having high-performance value and being non-responsive. This would be an advantage as many environments do not experience stressed conditions every season, or to the same level each season. A performance QTL that is independent of responsiveness would allow a lower risk alternative that would allow elite crop performance under both stressed and unstressed conditions as discussed by Telfer et al. (2021) and fits with the broad adaptation model proposed by Finlay and Wilkinson (1963).

Ten performance QTL were identified for grain yield, screenings, and TWT independent of any collocated responsiveness QTL (Fig. 1 and Supplementary Table 2). Grain yield QTL were identified on chromosome 2A (*QYld.agt-SG.2A*) and 7A (*QYld.agt-L2G.7A and QYld.agt-SG.7A*), with the Gladius parent providing the higher performing allele in each instance. *QYld.agt-SG.2A* on chromosome 2A occurs in region previously described for grain yield and thousand grain weight QTL by Tura et al. (2020) and Tsilo et al. (2010). On 7A, *QYld.agt-L2G.7A* occurs in a region where QTL for grain yield, kernel weight and TWT QTL have been previously reported (Cabral et al. 2018; Huang et al. 2003; Maphosa et al. 2014; Tura et al. 2020; Yu et al. 2018) including in heat stress studies (Bennett et al. 2012b; Guan et al. 2018; Mason et al. 2013; Pinto et al. 2016). Also, on 7A, *QYld.agt-SG.7A* occurred adjacent to a TWT performance QTL (*QTwt.agt-RG.7A*) identified in the RG population, with the Gladius parent being the source of the higher performance allele in both populations and for both traits. This is a region previously described by Pinto et al. (2016) and Mason et al. (2013) for thousand grain weight and grain yield, respectively, under late sown heat stress conditions. This region additionally has also previously been associated with thousand kernel weight (Huang et al. 2003) and test weight (Cabral et al. 2018) in studies investigating adaptation other than to heat stress conditions.

### Regions of responsiveness and their role in adaptation

Regions associated with responsiveness were identified separate from performance QTL, accounting for 42 of the QTL identified (Fig. 1 and Supplementary Table 2). Regions of responsiveness independent of performance QTL may provide opportunities to breed specific adaptation but would be more limited in their application and situations in which they confer an advantage. Under extreme stress conditions, such genomic regions may offer opportunities for improvements in adaption, especially if combined with other QTL for performance. When considering the framework proposed in Fig. 2h, this would be classified as a positive response and mean performance value.

To demonstrate, a region identified on chromosome 5A in the SG population where a grain yield QTL for responsiveness to grain fill number of days > 30 °C (*QYld.agt-SG.5A-2*) and grain fill number of days > 35 °C (*QYld.agt-SG.5A-1*) were found to collocate with the grain yield QTL responsive to growing season rainfall (*QYld.agt-SG.5A-3*) with the Scout parent providing the stress-adapted allele in each instance. This is a similar result to previous work by Tura et al. (2020), Zhang et al. (2018) and Tadesse et al. (2019) who reported QTL for grain yield and thousand grain weight in the same region. Additionally, Sharma et al. (2016) described this region for grain filling duration in heat stress conditions induced by delayed sowing.

In most instances where clusters of responsive QTL were found, there were QTL responsive to temperature and QTL responsive to growing season rainfall collocated. However, one example of collocated responsiveness QTL independent of growing season rainfall was identified for grain yield on chromosome 5B in the SM population (grain fill average maximum temperature—*QYld.agt-SM.5B-3* and grain fill number of days > 35 °C—*QYld.agt-SM.5B-4*). This is a region not previously described for adaptation to heat stress conditions during grain fill but has been associated with grain yield (Tura et al. 2020; Yu et al. 2018) and thousand grain weight (Tura et al. 2020). Each of the QTL reported by Tura et al. (2020) was found to have QTL-by-environment interactions, potentially identifying similar qualities for adaptation that have been determined to be responsive to changing temperature conditions in the current study. However, this region has been found to be associated with the number of leaves per seedling when heat stress conditions were imposed during the seedling stage (Maulana et al. 2018).

### Comparing field results to controlled environment evaluation of heat stress adaptation

The populations evaluated across multiple environments in this study to investigate adaptation to heat stress conditions were also evaluated previously in controlled environmental conditions by Telfer et al. (2021). In that study, adaptation to heat stress was investigated during grain filling by exposing plants to three consecutive eight hour days with an air temperature of 36 °C and a wind speed of 40 km h^−1^. QTL analysis was conducted to identify performance and responsiveness in a similar framework to the current study. When comparing the results of Telfer et al. (2021), 86 QTL from the current study were found to be collocated with 49 QTL identified in controlled environment conditions (excluding anthesis date QTL).

Key regions of crossover between that discussed by Telfer et al. (2021) and the current study include five performance QTL identified by Telfer et al. (2021) between 50.4 and 63.3 cM on chromosome 5B in the SM population, for thousand kernel weight, grain yield per spike, spikelet fertility, grain number per spike, and spikelet number per spike, where Mace was the source of the high-performance allele. In the current study, this is a region where numerous QTL were identified in the L2G, SM, MG, and SG populations. A grain yield performance QTL (*QYld.agt-SM.5B-1*) identified in the SM population clustered with screenings QTL responsive to numerous climatic co-variates and growing season rainfall. As with Telfer et al. (2021), Mace was the source of adaptation for stressed environments at this locus. Associations with grain yield have been previously identified in this region (Bennett et al. 2012b) in heat stress conditions, as well as thousand grain weight (Huang et al. 2003; Tsilo et al. 2010; Zhang et al. 2018).

In the current study, a grain yield performance QTL (*QYld.agt-SM.2B-2*) and TWT performance QTL (*QTwt.agt-SM.2B*) clustered with QTL for screenings and TWT responsive to various temperature co-variates and growing season rainfall. These QTL mapped similarly to six yield-related QTL identified in the SM and MG populations identified by Telfer et al. (2021). In both studies, Scout was responsible for the stress-adapted allele in the SM population and Gladius in the MG population where it was associated with increased thousand grain weight. This occurs in a region described by Hassan et al. (2018) for cytoplasmic membrane stability and grain number per spike (Sharma et al. 2016), both in heat stress conditions induced by delayed sowing, as well as grain yield and thousand grain weight (Cabral et al. 2018; Groos et al. 2003; Ramya et al. 2010; Tura et al. 2020; Yu et al. 2018).

Telfer et al. (2021) identified three performance QTL between 3.5 and 12.9 cM on chromosome 7A in the SG population for grain yield per spike, spikelet fertility, and grain number per spike where the adapted allele was sourced from the Scout parent. This corresponds to QTL identified in the current study between 13.3 and 23.1 cM in the SM population on chromosome 7A; a grain yield performance QTL (*QYld.agt-SM.7A*) and TWT QTL responsive to grain fill temperature co-variates, with the Mace allele being the allele conferring adaption to stressed conditions. The parents conferring the favourable allele were different for the QTL identified in the two different populations and studies. This makes interpretation of the favourable allele combination difficult and will require additional investigation. This is a region that has previously shown potential for adaptation to heat stress for grain yield in delayed sowing studies (Bennett et al. 2012b; Hassan et al. 2018; Pinto et al. 2016), as well as chlorophyll content (Talukder et al. 2014a).

In the current study, a grain yield performance QTL (*QYld.agt-L2G.7A*) on chromosome 7A in the L2G population was identified flanking a TWT QTL (*QTwt.agt-SG.7A*) responsive to grain fill average maximum temperature in the SG population. For both QTL, Gladius was the source of the elite or stress-adapted allele. These two QTL were identified on either side of two performance QTL identified by Telfer et al. (2021) for spikelet number per spike identified in the SG and L2G populations where Scout and AUS17840 provided the elite allele, respectively, in controlled environment conditions. This region has been described by Bennett et al. (2012b) and Mason et al. (2013) for improved grain yield under heat stress conditions induced by delayed sowing, as well as thousand grain weight (Bennett et al. 2012b; Guan et al. 2018), grain number per spike (Guan et al. 2018), and stay green (Mason et al. 2013). This region was also found to be important for grain yield determination in other adaptation studies (Tura et al. 2020) and thousand grain weight (Huang et al. 2004; Sun et al. 2008; Tsilo et al. 2010; Tura et al. 2020).

Telfer et al. (2021) identified two performance QTL on chromosome 6A (*QSfi.agt-SG.6A.2* and *QSfi.agt-L2G.6A*) that were flanked by three QTL identified in the current study (*QYld.agt-L2G.6A-1, QYld.agt-L2G.6A-2* and *QTwt.agt-RG.6A-2*). The populations involved within the two studies were not the same but did share Gladius as a common parent. The TWT response to stress is in repulsion to grain yield performance and responsive to temperature stress, while grain yield in the field appears to be linked positively to spikelet fertility in controlled environment conditions. This locus has been reported numerous times (Cui et al. 2014; Kuchel et al. 2007b; Mir et al. 2012; Pinto et al. 2016; Sun et al. 2008; Tura et al. 2020; Yu et al. 2018; Zhang et al. 2018) and is likely to be TaGW2 (Su et al. 2011) which confers a grain size advantage. Although TaGW2 was not mapped in the DH lines in this study, it was known to be segregating in some crosses, with Scout carrying the allele for large grain and Gladius the small grain allele. This suggests that the positive response in grain yield to heat stress seen in this study is linked to the TaGW2 allele for large grain.

Although some consistency between the field and in controlled environment condition studies can be demonstrated, there were more points of inconsistency. Likewise, links were found with other field studies conducted using delayed sowing, but these too were limited. This lack of commonality between the assay types suggests that the value of controlled environment studies is comparatively poor. It highlights the value of field-based genetic dissection of tolerance traits.

### Heat stress adaptation in the context of breeding objectives

As proposed by Telfer et al. (2021) and further developed here, heat stress adaptation should be considered as the combination of overall performance as well as responsiveness to stress. A definition that similarly could be applied to other stress conditions.

There are two types of QTL identified in this study: performance QTL which were found to be significant across the breadth of the environments observed, and responsive QTL that varied in expression in response to the abiotic stress encountered across the environments tested. QTL that combines positive performance with positive response to stress would be most desirable to breeders. While this study identified examples of a positive performance allele for grain yield also conferring a positive response to screenings or TWT QTL, these were rare and support the conclusion that breeding for heat stress tolerance is complex. Perhaps more realistically, a breeder seeking heat stress tolerance could target performance QTL that has been shown not to be responsive to heat stress. The results of this study certainly support this as a more achievable target with three QTL for grain yield identified that meet this criterion. Similarly, QTL that only show responsiveness to heat stress, and are not linked to performance, may offer opportunities to breed varieties that are specifically adapted to heat prone environments. This study highlights the importance of considering both performance and responsiveness when investigating tolerance to abiotic stress tolerance. Either in isolation may lead to incomplete or even worse, misguided conclusions regarding genetic selection strategy.

## Conclusions

This study demonstrates that adaptation to heat and drought can be assessed as the combination of performance and responsiveness to stress conditions. Furthermore, this two-dimensional framework for understanding and breeding for stress tolerance can be easily extended to other complex abiotic stresses. Loci for performance, with broad adaption across both stressed and unstressed environments in the absence of responsiveness, represent useful targets for breeders, with examples for grain yield identified in this study on chromosomes 2A and 7A. The responsiveness QTL identified offer opportunities to breed for specific adaption, including the grain yield locus found independent of performance on 5A that combines adaptation to heat stress during grain fill and drought stress. Finally, grain yield loci on 2B and 5B provide unique pathways to combine high performance with positive responsiveness to temperature and drought stress. These QTL, and others discussed in this study, present opportunities to further the development of diagnostic markers, firstly through validation in the populations used in this study and then wider validation in breeding populations.

Once validated these genetic regions represent opportunities for marker assisted selection by breeders. Additionally, the multi-environment framework used herein could be applied in the development of two-dimensional (performance and responsiveness) genomic prediction calibrations. This would enable the extension of current genomic selection principles, that are being increasingly used by breeding programs, to be extended into selection of adaptation to abiotic stress.

## Supplementary Information

Below is the link to the electronic supplementary material.

Supplementary Table 1. Consensus map and individual linkage maps for all populations affixed with RefSeq physical positions of QTL identified. Supplementary Table 2. All QTL identified by trait measured, QTL type (responsive or performance; if responsive the climatic co-variate to which responsiveness was found), interval position (cM) (consensus map position), the effect of each QTL, P-Value, LOD, and physical position (RefSeq). Supplementary Table 3. Genetic correlations between environments for each trait and population. (XLSX 913 KB)

## Data Availability

The datasets generated during the current study are available from the corresponding author on reasonable request.
